# *N*′-[(*E*)-4-Benz­yloxy-2-hy­droxy­benzyl­idene]-4-nitro­benzohydrazide monohydrate

**DOI:** 10.1107/S1600536812015401

**Published:** 2012-04-18

**Authors:** Bibitha Joseph, M. Sithambaresan, M. R. Prathapachandra Kurup

**Affiliations:** a Department of Applied Chemistry, Cochin University of Science and Technology, Kochi 682 022, India; b Department of Chemistry, Faculty of Science, Eastern University, Sri Lanka, Chenkalady, Sri Lanka

## Abstract

The title compound, C_21_H_17_N_3_O_5_·H_2_O, exists in the keto form with an *E* conformation with respect to the azomethine double bond. The twist angles between the aromatic rings are in the range 4.67 (10)–17.54 (10)°. A water mol­ecule of solvation is present in the lattice. A conventional intra­molecular O—H⋯N hydrogen bond increases the rigidity of the mol­ecule. Inter­molecular O—H⋯O, N—H⋯O and C—H⋯O hydrogen-bonding inter­actions establish a supra­molecular linkage among the mol­ecules in the crystal structure. There are also C—H⋯π inter­actions present.

## Related literature

For the biological and other applications of carbohydrazides, see: Lakshmi *et al.* (2011 ▶); Grande *et al.* (2007 ▶); Naseema *et al.* (2010 ▶). For the synthesis, see: Emmanuel *et al.* (2011 ▶). For related structures of carbohydrazides, see: Fun *et al.* (2008 ▶). For the keto form, see: Bakir & Brown (2002 ▶)*.* 
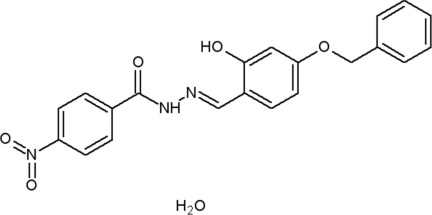


## Experimental

### 

#### Crystal data

C_21_H_17_N_3_O_5_·H_2_O
*M* *_r_* = 409.39
Monoclinic, 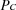

*a* = 4.6275 (7) Å
*b* = 6.5332 (11) Å
*c* = 31.856 (5) Å
β = 92.417 (4)°
*V* = 962.2 (3) Å^3^
*Z* = 2
Mo *K*α radiation
μ = 0.11 mm^−1^
*T* = 296 K
0.30 × 0.28 × 0.25 mm

#### Data collection

Bruker Kappa APEXII CCD diffractometer
Absorption correction: multi-scan (*SADABS*; Bruker, 2004 ▶) *T*_min_ = 0.969, *T*_max_ = 0.974
7388 measured reflections
1713 independent reflections
1663 reflections with *I* > 2σ(*I*)
*R*_int_ = 0.020

#### Refinement

*R*[*F* ^2^ > 2σ(*F* ^2^)] = 0.030
*wR*(*F* ^2^) = 0.088
*S* = 1.05
1713 reflections
288 parameters
7 restraints
H atoms treated by a mixture of independent and constrained refinement
Δρ_max_ = 0.14 e Å^−3^
Δρ_min_ = −0.16 e Å^−3^

### 

Data collection: *APEX2* (Bruker, 2004 ▶); cell refinement: *APEX2* and *SAINT* (Bruker, 2004 ▶); data reduction: *SAINT* and *XPREP* (Bruker, 2004 ▶); program(s) used to solve structure: *SHELXS97* (Sheldrick, 2008 ▶); program(s) used to refine structure: *SHELXL97* (Sheldrick, 2008 ▶); molecular graphics: *ORTEP-3* (Farrugia, 1997 ▶) and *DIAMOND* (Brandenburg, 2010 ▶); software used to prepare material for publication: *SHELXL97* and *publCIF* (Westrip, 2010 ▶).

## Supplementary Material

Crystal structure: contains datablock(s) global, I. DOI: 10.1107/S1600536812015401/fj2537sup1.cif

Structure factors: contains datablock(s) I. DOI: 10.1107/S1600536812015401/fj2537Isup2.hkl

Supplementary material file. DOI: 10.1107/S1600536812015401/fj2537Isup3.cml

Additional supplementary materials:  crystallographic information; 3D view; checkCIF report

## Figures and Tables

**Table 1:** Hydrogen-bond geometry (Å, °) *Cg* is the centroid of the C1–C6 ring

*D*—H⋯*A*	*D*—H	H⋯*A*	*D*⋯*A*	*D*—H⋯*A*
O4—H4*O*⋯O1*S*^i^	0.86 (2)	2.57 (4)	3.058 (3)	117 (4)
O4—H4*O*⋯N3	0.86 (2)	1.93 (3)	2.670 (2)	143 (4)
N2—H2*N*⋯O1*S*	0.86 (2)	2.01 (2)	2.855 (3)	166 (3)
O1*S*—H2*S*⋯O4^ii^	0.87 (2)	2.01 (2)	2.860 (3)	165 (4)
O1*S*—H1*S*⋯O3^iii^	0.88 (2)	1.80 (2)	2.676 (3)	175 (5)
C14—H14⋯O1*S*	0.93	2.37	3.184 (2)	146
C21—H21⋯O1*S*	0.93	2.43	3.325 (3)	161
C7—H7*B*⋯*Cg*^iv^	0.97	2.69	3.472 (2)	138

Symmetry codes: (i) 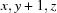; (ii) 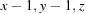; (iii) 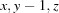; (iv) 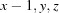.

**Fig. 1.**
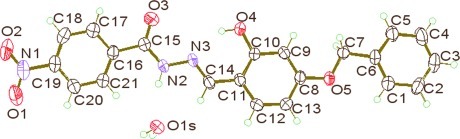
ORTEP view of the molecular structure of the title compound, along with the atom-labelling, drawn with 50% probability displacement ellipsoids.

**Fig. 2.**
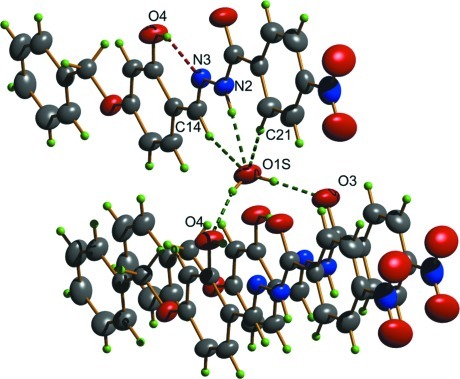
A view of the conventional and non-conventional hydrogen bonding interactions

**Fig. 3.**
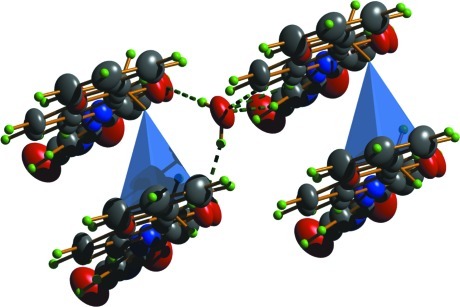
Graphical representation showing C–H···π interaction in the crystal structure of C21H17N3O5.H2O.

**Fig. 4.**
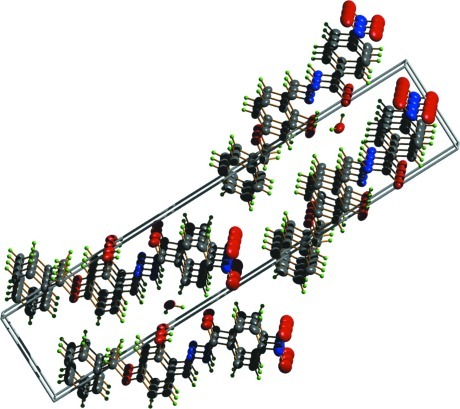
Packing arrangement of molecules along a axis.

## supplementary crystallographic information

### Comment

There is growing interest in the structural features of carbohydrazides as they show a wide range of biological activities, with potential uses in antibacterial, antifungal and anticancer studies (Lakshmi *et al.*, 2011; Grande *et al.*, 2007). Hydrazones and their metal complexes have found applications in chemical processes like non linear optics, sensors *etc*. (Naseema *et al.*, 2010).

The title compound *N*'-{(*E*)-[4-(benzyloxy)-2-hydroxyphenyl]methylidene}-4-nitrobenzohydrazide hydrate is found to exist in the *E* configuration with respect to N3=C14 bond. A perspective view of the molecular structure of the title compound, along with the atom-labeling is shown in Fig. 1. The bond length of C15=O3 [1.229 (2) Å] shows a significant double-bond character (Fun *et al.*., 2008) indicating that the molecule exists in the keto form in the solid state (Bakir & Brown, 2002) and the dihedral angles between the aromatic rings are in the range of 4.67 (10) -17.54 (10)°.

The lattice water molecule plays an essential role in packing of the molecules forming conventional and non-conventional hydrogen bonds between the carbohydrazide and water molecules (Fig. 2). A C–H···π interaction is also observed in the crystal structure between one of the H atoms attached to the C7 carbon atom and the phenyl ring of the adjacent molecule in the crystal system (Fig. 3). Two types of very weak π–π interactions also present with a shortest centroid-centroid distance of 4.9302 (14) Å. In crystal packing, the parallel arrangement of the molecules along *a* axis is shown in Fig. 4.

### Experimental

The title compound was prepared by adapting a reported procedure (Emmanuel *et al.*, 2011) by refluxing a mixture of methanolic solutions of 4-nitrobenzohydrazide (0.181 g, 1 mmol) and 4-(benzyloxy)-2-hydroxybenzaldehyde (0.228 g, 1 mmol) for 4 h. The formed crystals were collected, washed with few drops of methanol and dried over P_4_O_10_*in vacuo*. Single crystals of the title compound suitable for X-ray analysis were obtained by slow evaporation from its methanolic solution.

### Refinement

All H atoms on C were placed in calculated positions, guided by difference maps, with C—H bond distances 0.93–0.97 Å. H atoms were assigned as *U*_iso_=1.2 *U*_eq_. N2—H2N and O4—H4O (0.86 Å) H atoms were located from difference maps and restrained using *DFIX* instructions. The O1S—H1S and O1S—H2S (0.86 Å) H atoms of the water molecule is also located from difference maps and restrained using *DFIX* and *DANG* instructions.

In the absence of significant anomalous scattering effects Friedel pairs have been merged.

### Crystal data

**Table:**

C_21_H_17_N_3_O_5_·H_2_O	*F*(000) = 428.0
*M**_r_* = 409.39	*D*_x_ = 1.413 Mg m^−^^3^
Monoclinic, *P**c*	Mo *K*α radiation, λ = 0.71073 Å
Hall symbol: P -2yc	Cell parameters from 4698 reflections
*a* = 4.6275 (7) Å	θ = 3.1–28.2°
*b* = 6.5332 (11) Å	µ = 0.11 mm^−^^1^
*c* = 31.856 (5) Å	*T* = 296 K
β = 92.417 (4)°	Block, yellow
*V* = 962.2 (3) Å^3^	0.30 × 0.28 × 0.25 mm
*Z* = 2	

### Data collection

**Table:**

Bruker Kappa APEXII CCD diffractometer	1713 independent reflections
Radiation source: fine-focus sealed tube	1663 reflections with *I* > 2σ(*I*)
Graphite monochromator	*R*_int_ = 0.020
ω and φ scan	θ_max_ = 25.0°, θ_min_ = 3.1°
Absorption correction: multi-scan (*SADABS*; Bruker, 2004)	*h* = −3→5
*T*_min_ = 0.969, *T*_max_ = 0.974	*k* = −7→7
7388 measured reflections	*l* = −37→37

### Refinement

**Table:**

Refinement on *F*^2^	Secondary atom site location: difference Fourier map
Least-squares matrix: full	Hydrogen site location: inferred from neighbouring sites
*R*[*F*^2^ > 2σ(*F*^2^)] = 0.030	H atoms treated by a mixture of independent and constrained refinement
*wR*(*F*^2^) = 0.088	*w* = 1/[σ^2^(*F*_o_^2^) + (0.0687*P*)^2^ + 0.0484*P*] where *P* = (*F*_o_^2^ + 2*F*_c_^2^)/3
*S* = 1.05	(Δ/σ)_max_ = 0.011
1713 reflections	Δρ_max_ = 0.14 e Å^−^^3^
288 parameters	Δρ_min_ = −0.16 e Å^−^^3^
7 restraints	Extinction correction: *SHELXL97* (Sheldrick, 2008), Fc^*^=kFc[1+0.001xFc^2^λ^3^/sin(2θ)]^-1/4^
Primary atom site location: structure-invariant direct methods	Extinction coefficient: 0.021 (7)

### Special details

**Table:**

Geometry. All e.s.d.'s (except the e.s.d. in the dihedral angle between two l.s. planes) are estimated using the full covariance matrix. The cell e.s.d.'s are taken into account individually in the estimation of e.s.d.'s in distances, angles and torsion angles; correlations between e.s.d.'s in cell parameters are only used when they are defined by crystal symmetry. An approximate (isotropic) treatment of cell e.s.d.'s is used for estimating e.s.d.'s involving l.s. planes.
Refinement. Refinement of *F*^2^ against ALL reflections. The weighted *R*-factor *wR* and goodness of fit *S* are based on *F*^2^, conventional *R*-factors *R* are based on *F*, with *F* set to zero for negative *F*^2^. The threshold expression of *F*^2^ > σ(*F*^2^) is used only for calculating *R*-factors(gt) *etc*. and is not relevant to the choice of reflections for refinement. *R*-factors based on *F*^2^ are statistically about twice as large as those based on *F*, and *R*- factors based on ALL data will be even larger.

### Fractional atomic coordinates and isotropic or equivalent isotropic displacement parameters (Å^2^)

**Table:**

	*x*	*y*	*z*	*U*_iso_*/*U*_eq_	
O1	−0.5580 (7)	0.1667 (5)	0.47190 (9)	0.0986 (10)	
O2	−0.5671 (7)	0.4470 (5)	0.50699 (9)	0.0960 (9)	
O3	0.3371 (5)	0.9071 (3)	0.37071 (7)	0.0673 (6)	
O4	0.8452 (4)	1.0067 (3)	0.26751 (6)	0.0537 (5)	
O5	1.4446 (4)	0.8132 (2)	0.15654 (5)	0.0428 (4)	
O1S	0.3303 (5)	0.1942 (3)	0.31076 (7)	0.0596 (5)	
N1	−0.4868 (6)	0.3450 (5)	0.47795 (8)	0.0623 (7)	
N2	0.3751 (4)	0.6173 (3)	0.33257 (6)	0.0380 (4)	
N3	0.5600 (4)	0.7064 (3)	0.30472 (6)	0.0370 (4)	
C1	1.8112 (6)	0.8824 (4)	0.09200 (9)	0.0507 (6)	
H1	1.7528	0.7502	0.0983	0.061*	
C2	2.0020 (6)	0.9151 (5)	0.05996 (9)	0.0593 (8)	
H2	2.0703	0.8044	0.0449	0.071*	
C3	2.0894 (6)	1.1089 (5)	0.05048 (9)	0.0589 (8)	
H3	2.2186	1.1292	0.0293	0.071*	
C4	1.9873 (6)	1.2742 (5)	0.07214 (9)	0.0600 (7)	
H4	2.0454	1.4061	0.0654	0.072*	
C5	1.7976 (6)	1.2430 (4)	0.10394 (9)	0.0508 (6)	
H5	1.7282	1.3546	0.1186	0.061*	
C6	1.7094 (4)	1.0458 (4)	0.11423 (7)	0.0377 (5)	
C7	1.5050 (5)	1.0238 (4)	0.14926 (7)	0.0391 (5)	
H7A	1.5901	1.0846	0.1747	0.047*	
H7B	1.3264	1.0957	0.1421	0.047*	
C8	1.2534 (4)	0.7701 (4)	0.18637 (7)	0.0355 (5)	
C9	1.1417 (5)	0.9148 (3)	0.21297 (7)	0.0371 (5)	
H9	1.1963	1.0513	0.2108	0.044*	
C10	0.9484 (4)	0.8566 (4)	0.24291 (7)	0.0345 (5)	
C11	0.8636 (4)	0.6529 (4)	0.24699 (7)	0.0346 (5)	
C12	0.9782 (5)	0.5107 (4)	0.21932 (8)	0.0430 (5)	
H12	0.9233	0.3742	0.2212	0.052*	
C13	1.1688 (5)	0.5665 (4)	0.18961 (8)	0.0434 (5)	
H13	1.2415	0.4686	0.1717	0.052*	
C14	0.6665 (5)	0.5843 (4)	0.27812 (7)	0.0388 (5)	
H14	0.6156	0.4467	0.2788	0.047*	
C15	0.2747 (5)	0.7263 (4)	0.36431 (8)	0.0405 (5)	
C16	0.0762 (5)	0.6172 (4)	0.39327 (7)	0.0382 (5)	
C17	−0.0090 (6)	0.7250 (4)	0.42798 (8)	0.0522 (6)	
H17	0.0583	0.8576	0.4326	0.063*	
C18	−0.1943 (6)	0.6370 (5)	0.45600 (8)	0.0567 (7)	
H18	−0.2526	0.7092	0.4793	0.068*	
C19	−0.2898 (5)	0.4403 (4)	0.44837 (7)	0.0452 (6)	
C20	−0.2096 (6)	0.3294 (4)	0.41418 (9)	0.0524 (6)	
H20	−0.2769	0.1967	0.4098	0.063*	
C21	−0.0266 (6)	0.4198 (4)	0.38649 (8)	0.0494 (6)	
H21	0.0285	0.3474	0.3630	0.059*	
H1S	0.325 (10)	0.105 (6)	0.3313 (11)	0.096 (14)*	
H2S	0.169 (6)	0.160 (6)	0.2975 (12)	0.099 (14)*	
H2N	0.336 (6)	0.489 (3)	0.3287 (9)	0.046 (7)*	
H4O	0.730 (7)	0.958 (7)	0.2853 (11)	0.090 (13)*	

### Atomic displacement parameters (Å^2^)

**Table:**

	*U*^11^	*U*^22^	*U*^33^	*U*^12^	*U*^13^	*U*^23^
O1	0.123 (2)	0.097 (2)	0.0791 (17)	−0.0492 (18)	0.0467 (15)	−0.0001 (15)
O2	0.1081 (19)	0.108 (2)	0.0771 (16)	−0.0053 (17)	0.0631 (14)	−0.0006 (15)
O3	0.1030 (17)	0.0439 (11)	0.0571 (11)	−0.0205 (10)	0.0283 (10)	−0.0053 (9)
O4	0.0609 (11)	0.0409 (10)	0.0623 (11)	−0.0109 (8)	0.0384 (9)	−0.0097 (8)
O5	0.0446 (8)	0.0385 (9)	0.0470 (9)	0.0009 (8)	0.0217 (7)	0.0040 (7)
O1S	0.0790 (14)	0.0383 (9)	0.0638 (12)	−0.0155 (9)	0.0283 (10)	−0.0050 (9)
N1	0.0571 (14)	0.0820 (19)	0.0491 (12)	−0.0043 (13)	0.0172 (11)	0.0143 (13)
N2	0.0434 (10)	0.0318 (10)	0.0400 (10)	−0.0066 (8)	0.0150 (8)	0.0050 (8)
N3	0.0357 (10)	0.0364 (10)	0.0398 (10)	−0.0075 (8)	0.0127 (7)	0.0060 (8)
C1	0.0523 (14)	0.0453 (14)	0.0560 (15)	−0.0009 (12)	0.0202 (12)	0.0008 (11)
C2	0.0609 (17)	0.065 (2)	0.0547 (15)	0.0085 (14)	0.0296 (13)	−0.0029 (13)
C3	0.0500 (14)	0.081 (2)	0.0477 (14)	0.0040 (14)	0.0227 (11)	0.0143 (15)
C4	0.0598 (16)	0.0594 (17)	0.0624 (17)	−0.0058 (14)	0.0212 (13)	0.0237 (15)
C5	0.0517 (14)	0.0444 (14)	0.0579 (14)	0.0025 (11)	0.0195 (11)	0.0093 (12)
C6	0.0303 (10)	0.0455 (12)	0.0378 (11)	0.0008 (10)	0.0089 (9)	0.0078 (10)
C7	0.0378 (11)	0.0382 (12)	0.0420 (12)	0.0016 (10)	0.0118 (9)	0.0024 (10)
C8	0.0334 (11)	0.0391 (12)	0.0346 (10)	0.0020 (9)	0.0097 (8)	0.0037 (9)
C9	0.0390 (11)	0.0304 (11)	0.0426 (12)	−0.0034 (9)	0.0106 (9)	0.0016 (9)
C10	0.0353 (11)	0.0327 (12)	0.0362 (11)	−0.0026 (9)	0.0100 (8)	−0.0011 (9)
C11	0.0312 (10)	0.0356 (12)	0.0374 (11)	−0.0039 (9)	0.0066 (9)	0.0019 (9)
C12	0.0457 (12)	0.0300 (11)	0.0542 (14)	−0.0021 (10)	0.0139 (10)	0.0002 (10)
C13	0.0454 (12)	0.0365 (12)	0.0497 (12)	0.0018 (10)	0.0174 (10)	−0.0052 (10)
C14	0.0382 (11)	0.0338 (12)	0.0452 (12)	−0.0049 (9)	0.0102 (10)	0.0073 (9)
C15	0.0459 (12)	0.0373 (12)	0.0389 (11)	−0.0050 (10)	0.0095 (9)	0.0028 (10)
C16	0.0393 (11)	0.0419 (12)	0.0341 (11)	0.0032 (10)	0.0082 (9)	0.0054 (9)
C17	0.0611 (16)	0.0511 (15)	0.0455 (13)	−0.0088 (13)	0.0140 (11)	−0.0051 (12)
C18	0.0648 (16)	0.0659 (17)	0.0409 (13)	−0.0006 (14)	0.0201 (11)	−0.0074 (13)
C19	0.0388 (12)	0.0614 (17)	0.0362 (12)	0.0002 (11)	0.0100 (9)	0.0102 (11)
C20	0.0569 (15)	0.0443 (14)	0.0575 (15)	−0.0059 (12)	0.0198 (12)	0.0053 (12)
C21	0.0580 (15)	0.0452 (14)	0.0468 (13)	−0.0061 (12)	0.0232 (11)	−0.0028 (11)

### Geometric parameters (Å, º)

**Table:**

O1—N1	1.224 (4)	C6—C7	1.500 (3)
O2—N1	1.211 (4)	C7—H7A	0.9700
O3—C15	1.231 (3)	C7—H7B	0.9700
O4—C10	1.355 (3)	C8—C9	1.384 (3)
O4—H4O	0.86 (2)	C8—C13	1.392 (4)
O5—C8	1.355 (3)	C9—C10	1.388 (3)
O5—C7	1.425 (3)	C9—H9	0.9300
O1S—H1S	0.88 (2)	C10—C11	1.395 (3)
O1S—H2S	0.873 (19)	C11—C12	1.400 (3)
N1—C19	1.476 (3)	C11—C14	1.446 (3)
N2—C15	1.336 (3)	C12—C13	1.370 (3)
N2—N3	1.386 (3)	C12—H12	0.9300
N2—H2N	0.863 (19)	C13—H13	0.9300
N3—C14	1.277 (3)	C14—H14	0.9300
C1—C6	1.375 (4)	C15—C16	1.508 (3)
C1—C2	1.394 (4)	C16—C17	1.383 (4)
C1—H1	0.9300	C16—C21	1.388 (4)
C2—C3	1.367 (5)	C17—C18	1.388 (4)
C2—H2	0.9300	C17—H17	0.9300
C3—C4	1.376 (5)	C18—C19	1.377 (4)
C3—H3	0.9300	C18—H18	0.9300
C4—C5	1.383 (4)	C19—C20	1.372 (4)
C4—H4	0.9300	C20—C21	1.381 (4)
C5—C6	1.394 (3)	C20—H20	0.9300
C5—H5	0.9300	C21—H21	0.9300
			
C10—O4—H4O	111 (3)	C8—C9—H9	119.9
C8—O5—C7	116.93 (17)	C10—C9—H9	119.9
H1S—O1S—H2S	98 (3)	O4—C10—C9	116.9 (2)
O2—N1—O1	123.7 (3)	O4—C10—C11	122.0 (2)
O2—N1—C19	118.2 (3)	C9—C10—C11	121.2 (2)
O1—N1—C19	118.0 (3)	C10—C11—C12	117.30 (19)
C15—N2—N3	120.28 (19)	C10—C11—C14	123.2 (2)
C15—N2—H2N	123 (2)	C12—C11—C14	119.5 (2)
N3—N2—H2N	117 (2)	C13—C12—C11	122.0 (2)
C14—N3—N2	115.35 (19)	C13—C12—H12	119.0
C6—C1—C2	120.0 (3)	C11—C12—H12	119.0
C6—C1—H1	120.0	C12—C13—C8	119.8 (2)
C2—C1—H1	120.0	C12—C13—H13	120.1
C3—C2—C1	120.4 (3)	C8—C13—H13	120.1
C3—C2—H2	119.8	N3—C14—C11	122.3 (2)
C1—C2—H2	119.8	N3—C14—H14	118.9
C2—C3—C4	120.3 (2)	C11—C14—H14	118.9
C2—C3—H3	119.8	O3—C15—N2	123.4 (2)
C4—C3—H3	119.8	O3—C15—C16	119.8 (2)
C3—C4—C5	119.6 (3)	N2—C15—C16	116.8 (2)
C3—C4—H4	120.2	C17—C16—C21	119.4 (2)
C5—C4—H4	120.2	C17—C16—C15	116.8 (2)
C4—C5—C6	120.6 (3)	C21—C16—C15	123.9 (2)
C4—C5—H5	119.7	C16—C17—C18	120.5 (3)
C6—C5—H5	119.7	C16—C17—H17	119.7
C1—C6—C5	119.1 (2)	C18—C17—H17	119.7
C1—C6—C7	123.3 (2)	C19—C18—C17	118.4 (2)
C5—C6—C7	117.6 (2)	C19—C18—H18	120.8
O5—C7—C6	110.37 (19)	C17—C18—H18	120.8
O5—C7—H7A	109.6	C20—C19—C18	122.5 (2)
C6—C7—H7A	109.6	C20—C19—N1	118.6 (2)
O5—C7—H7B	109.6	C18—C19—N1	118.9 (2)
C6—C7—H7B	109.6	C19—C20—C21	118.4 (2)
H7A—C7—H7B	108.1	C19—C20—H20	120.8
O5—C8—C9	124.0 (2)	C21—C20—H20	120.8
O5—C8—C13	116.4 (2)	C20—C21—C16	120.9 (2)
C9—C8—C13	119.6 (2)	C20—C21—H21	119.6
C8—C9—C10	120.1 (2)	C16—C21—H21	119.6
			
C15—N2—N3—C14	173.2 (2)	O5—C8—C13—C12	179.4 (2)
C6—C1—C2—C3	0.1 (5)	C9—C8—C13—C12	−0.6 (4)
C1—C2—C3—C4	−0.8 (5)	N2—N3—C14—C11	−179.85 (19)
C2—C3—C4—C5	0.7 (5)	C10—C11—C14—N3	−1.3 (3)
C3—C4—C5—C6	0.1 (4)	C12—C11—C14—N3	178.5 (2)
C2—C1—C6—C5	0.6 (4)	N3—N2—C15—O3	0.1 (4)
C2—C1—C6—C7	−179.7 (3)	N3—N2—C15—C16	−179.66 (19)
C4—C5—C6—C1	−0.7 (4)	O3—C15—C16—C17	−4.8 (4)
C4—C5—C6—C7	179.6 (2)	N2—C15—C16—C17	175.0 (2)
C8—O5—C7—C6	−177.51 (18)	O3—C15—C16—C21	174.1 (3)
C1—C6—C7—O5	2.1 (3)	N2—C15—C16—C21	−6.2 (3)
C5—C6—C7—O5	−178.1 (2)	C21—C16—C17—C18	0.3 (4)
C7—O5—C8—C9	−7.7 (3)	C15—C16—C17—C18	179.2 (2)
C7—O5—C8—C13	172.3 (2)	C16—C17—C18—C19	0.2 (4)
O5—C8—C9—C10	−179.4 (2)	C17—C18—C19—C20	−0.3 (4)
C13—C8—C9—C10	0.5 (3)	C17—C18—C19—N1	180.0 (2)
C8—C9—C10—O4	−179.2 (2)	O2—N1—C19—C20	−176.7 (3)
C8—C9—C10—C11	0.2 (3)	O1—N1—C19—C20	3.9 (4)
O4—C10—C11—C12	178.5 (2)	O2—N1—C19—C18	3.0 (4)
C9—C10—C11—C12	−0.9 (3)	O1—N1—C19—C18	−176.4 (3)
O4—C10—C11—C14	−1.6 (3)	C18—C19—C20—C21	−0.1 (4)
C9—C10—C11—C14	179.0 (2)	N1—C19—C20—C21	179.6 (2)
C10—C11—C12—C13	0.8 (3)	C19—C20—C21—C16	0.6 (4)
C14—C11—C12—C13	−179.1 (2)	C17—C16—C21—C20	−0.7 (4)
C11—C12—C13—C8	−0.1 (4)	C15—C16—C21—C20	−179.6 (2)

### Hydrogen-bond geometry (Å, º)

Cg is the centroid of the C1–C6 ring

**Table:**

*D*—H···*A*	*D*—H	H···*A*	*D*···*A*	*D*—H···*A*
O4—H4*O*···O1*S*^i^	0.86 (2)	2.57 (4)	3.058 (3)	117 (4)
O4—H4*O*···N3	0.86 (2)	1.93 (3)	2.670 (2)	143 (4)
N2—H2*N*···O1*S*	0.86 (2)	2.01 (2)	2.855 (3)	166 (3)
O1*S*—H2*S*···O4^ii^	0.87 (2)	2.01 (2)	2.860 (3)	165 (4)
O1*S*—H1*S*···O3^iii^	0.88 (2)	1.80 (2)	2.676 (3)	175 (5)
C14—H14···O1*S*	0.93	2.37	3.184 (2)	146
C21—H21···O1*S*	0.93	2.43	3.325 (3)	161
C7—H7*B*···*Cg*^iv^	0.97	2.69	3.472 (2)	138

Symmetry codes: (i) *x*, *y*+1, *z*; (ii) *x*−1, *y*−1, *z*; (iii) *x*, *y*−1, *z*; (iv) *x*−1, *y*, *z*.
